# Current pattern of Ponderal Indices of term small-for-gestational age in a population of Nigerian babies

**DOI:** 10.1186/1471-2431-13-110

**Published:** 2013-07-23

**Authors:** Olubanke R Oluwafemi, Fidelis O Njokanma, Elizabeth A Disu, Tinuade A Ogunlesi

**Affiliations:** 1 Department of Paediatrics, Mother and Child Hospital, Akure, Ondo State, Nigeria; 2 Department of Paediatrics and Child Health, Lagos State University Teaching Hospital, Ikeja, Lagos, Nigeria; 3 Department of Paediatrics, Olabisi Onabanjo University Teaching Hospital, Sagamu, Ogun State, Nigeria

**Keywords:** Small-for-gestational age, Intrauterine growth, Ponderal Index

## Abstract

**Background:**

Small-for-gestational age (SGA) newborns constitute a special group of neonates who may have suffered varying degrees of intrauterine insults and deprivation. Variations in birth weight, length and Ponderal Index (PI) depend on the type and degree of intrauterine insults the babies were exposed to. The objective of the study was to determine the current prevalence of term SGA births in a Nigerian Tertiary Hospital and the current pattern of Ponderal Indices among term SGA in a population of Nigerian babies.

**Methods:**

Subjects comprised of consecutive term singleton mother-baby pairs in the first 24 hours of life. It was a cross sectional study. The anthropometric parameters of each baby were recorded and the PI was also determined.

**Results:**

Out of 1,052 live births during the study period (September to December, 2009), 825 were term, singleton babies. Five hundred and eight-one babies (70.4%) fall into the upper socio-economic classes 1 and II, 193 (23.4%) in the middle class and 51 (6.2%) were of the lower classes IV and V. None of the mothers indicated ingestion of alcohol or smoking of cigarette. Fifty-nine babies (7.2%) were small-for gestational age (SGA). Of the 59 SGA subjects, 26 (44.1%) were symmetrical SGA while 33 (55.9%) were asymmetrical SGA. There was no significant sex or socioeconomic predilection for either symmetrical or asymmetrical growth (p = 0.59, 0.73 respectively).

**Conclusion:**

The findings showed that proportionality in SGA fetuses is a continuum, with the PI depending on the duration of intrauterine insult and the extent of its effects on weight and length before delivery.

## Background

The term ‘small- for-gestational age’ (SGA) describes a baby whose birth weight is less than the 10^th^ percentile or more than two standard deviations below the mean for age and sex [1] or less than 2.5 kg for a term neonate. The prevalence of SGA in the United States is 10% with a higher incidence recorded among the black population [2]. The prevalence in Sweden is 2.8 to 3.4 percent [3] while in the United Kingdom it is 4.1% [4]. In Nigeria, prevalence rates of 12.8% and 12.01% were recorded in Sagamu [5] and Ilesa [6] respectively.

SGA babies may be symmetrically or asymmetrically small. Longstanding intra-uterine insults, particularly those occurring during the phase of early fetal cellular hyperplasia will have proportionately small occipito-frontal circumference (OFC), length and weight [7]. These infants are referred to as symmetrical SGA babies [7,8]. Consequent upon the reduction in growth parameters in these babies, particularly in the weight and length, the Ponderal Index (PI) (weight in grams x 100/length in cm [3]) may be apparently normal. Asymmetric SGA on the other hand, results from acute insults on the fetus frequently occurring during the phase of cellular hypertrophy in the third trimester [7-9]. The consequence is a relatively normal OFC, some reduction in length but a more profound reduction of the rate of weight accretion [7]. As a result, the PI may be low [7]. This is thought to be due to the redistribution of the fetal blood flow preferentially to vital organs such as the brain, hence the “head-sparing effect.” [7] In clinical practice, emphasis is often placed on body proportions in assessing growth and nutritional status. One of the most favoured proportions used in neonatal care is the PI or the weight-length ratio [7], which helps to quantify the weight loss [7]. It finds use in sub-classifying SGA babies according to the proportionality of growth restriction. It is important to distinguish between symmetrical and asymmetrical SGA because the immediate neonatal morbidities and long term outcomes differ [7]. The symmetrical SGA babies have higher tendencies to suffer more morbidities while the asymmetrical ones may have higher incidence of perinatal mortality [7]. This helps in making comprehensive perinatal and neonatal care plans. Thus, preventive measures could be applied where feasible. The concept of PI was first introduced by Campbell and Thoms in 1977. [10] Therefore, the current study is aimed at determining the prevalence of term small-for-gestational age babies delivered in Lagos State University Teaching Hospital (LASUTH), Ikeja, Lagos, Nigeria and also to describe the pattern of Ponderal Indices.

## Methods

This study was carried out in the Lagos State University Teaching Hospital (LASUTH), Ikeja, Lagos, Nigeria. Lagos, the commercial capital city of Nigeria, has cosmopolitan characteristics. It is one of the cities located in the coastal part of South-Western Nigeria with rapid urbanization and industrialization. The city is situated at the sea level [11]. Socio-economic status of the residents vary depending on the level of industrialization in the various parts of the city. The LASUTH is located right within the administrative capital of Lagos State of Nigeria. The hospital serves as a referral centre for private and public primary and secondary health institutions in both Lagos and the neighboring Ogun state. The average delivery rate in the maternity section of the hospital is 4000 babies per annum, with term babies accounting for approximately 90%. There is no restriction on the type of cases admitted into this facility. Thus, women of all socioeconomic classes patronize the hospital.

The study was hospital based and cross sectional in design. The study took place between September and December 2009. The subjects consisted of consecutive mother-baby pairs who fulfilled set study criteria. Inclusion criteria included term newborn babies, singleton birth of consenting mothers while exclusion criterion babies with gross congenital abnormality.

The methods involved physical examination of consecutive term (≥ 37 to 42weeks gestation) singleton babies during the first 24 hours of life, including measurement of weight, length and OFC, using standard defined techniques. Each baby was weighed using the *RGZ-20* weighing scale. The scale records weights in grams to the nearest 25 g. It was adjusted for zero error before each reading. Other measures taken to ensure reliability of results included weekly standardization of the weighing scale, using known weights.

Length was measured in centimeters to one decimal place using a metal anthropometric linear rule fixed to a horizontal flat board. The board had a fixed head plate at the zero end of the metal rule and a sliding foot plate. An assistant held the baby in position, making sure that the head is flushed with the fixed head board, the eye-sockets are in the same vertical plane as the external auditory meati, the back is flat on the board and the knees are straight. The investigator then slides the foot plate to gently touch the sole of the baby’s foot and read off the length [8,12].

The OFC was measured by the investigator to the nearest 0.1cm with a non stretchable tape using the glabella and the tip of the occiput as the landmarks [8,12].

PI was derived from weight and length (weight in g x 100/length in cm^3^) [12].

The gestational age was determined calculating from the first day of last maternal menstrual period. This was corroborated by the scores obtained by using methods described by Ballard *et al*. [13]. Where the difference between both techniques was more than two weeks, the gestational age obtained by using the New Ballard score was upheld and recorded.

SGA babies were those with birth weight below the 10^th^ percentile of the Lubchenco chart [12]. Symmetrical ones also had length measurements below the 10^th^ percentile of the Lubchenco chart, while the length of asymmetrical ones was above the 10^th^ percentile. Maternal data; age, parity, anthropometry (weight and height), level of education, occupation, inter-pregnancy interval, blood pressure, social habits such as smoking and alcoholism, and type of illness during pregnancy were obtained through direct interview as well as from clinical records. The maternal weight was taken using the *RGZ-160 (MA-DONAX ENGLAND)* weight and height scale. The scale records weights in kilograms to the nearest 50 g and it also has a sliding meter rule attached to it for the purpose of height measurement. The height ranged from 70 to 190 cm to the nearest 0.5 cm. The weighing scale was adjusted for zero error before each weight reading and while the patient was still standing on it without her shoes on, the meter rule is adjusted to touch her vertex and the value was read off. The body mass index (BMI) was then calculated using the formular; weight (kg) / height (m^2^) while the socioeconomic class was assessed based on occupation and educational levels as described by Oyedeji [14].

Ethical clearance was obtained from the hospital’s Research/Ethics Committee and written informed consent was taken from mothers of subjects at contact.

The minimum sample size ‘N’ for the study was based on the incidence of SGA delivery being one of the major outcome criteria and was estimated from the formula: [15,16]

$$N=4\;{\left(\text{Zcrit}\right)}^{2}p\;\left(1-p\right)÷D^{2}$$

Where :

‘Zcrit’ is the fraction of area under a normal distribution curve covered by two standard deviation (2SD) on either sides of the mean. It is equal to 1.96 in a two-tailed test.

‘p’ is the pre-study estimate or known prevalence of the variable under study, which was taken as 12.8 percent (0.128), based on an earlier report by Njokanma and Sule-Odu [5].

‘D’ is the absolute tolerable sampling error. In this study, it was fixed at 5 percent (0.05) [5].

Substituting these figures into the formula,

$$‘N’=4{\left(1.96\right)}^{2}x\;0.128\;x\;0.872÷0.05^{2}=686.$$

In order to accommodate unforeseen errors in completing the study questionnaire, additional 20% (138 subjects) were studied. Therefore the set sample size for this study was 825.

The data was analyzed using Microsoft Excel program and SPSS version 16.0. Descriptive and inferential statistics were applied in the course of analysis. *Pearson’s Chi-square test* was used to assess relationships between categorical variable. The *p*-value less than 0.05 defined statistical significance (95% confidence level).

## Results

Eight hundred and twenty-five term singleton babies were examined, 59 (7.2%) were SGA. Of the 59 SGA babies, 26 (44.1%) were symmetrically small and 33 (55.9%) were asymmetrically small.

In Table 1, there was no significant sex or socioeconomic predilection for either symmetrical or asymmetrical growth (p = 0.59, 0.73 respectively). Symmetrical SGA babies had significantly smaller mean length and OFC than their asymmetrical counterparts (p = 0.000, 0.013) respectively. On the other hand, the symmetrical SGA babies had a significantly higher mean PI (p = 0.000).

**Table 1:** Sub-classification of small-for-gestational age babies according to Sex, socioeconomic index and anthropometric indices

	**Symmetrical**	**Asymmetrical**	**All**		
	**N (%)**	**N (%)**	**N (%)**	***χ***^**2**^	**p**
**Sex**					
Male	10 (40.0)	15 (60.0)	25 (100.0)		
Female	16 (47.1)	18 (52.9)	34 (100.0)		
Total	26 (44.1)	33 (55.9)	59 (100.0)	0.29	0.59
**Socioeconomic categories**					
Upper (I and II)	13 (41.9)	18 (58.1)	31 (100.0)		
Others (III, IV,V)	13 (46.4)	15 (53.6)	28 (100.0)		
Total	26 (44.1)	33 (55.9)	59 (100.0)	0.12	0.73
**Anthropometric indices**					
	**Mean ± SD**	**Mean ± SD**	**Mean ± SD**	**t**	**p**
Birth weight g	2287 ± 227	2377 ± 245	2337 ± 240	1.46	0.15
Length cm	44.2 ± 1.24	47.4 ± 1.73	46.0 ± 2.19	8.27	0.000
OFC cm	32.6 ± 0.99	33.3 ± 1.10	33.0 ± 1.10	2.57	0.013
PI g/cm^3^	2.65 ± 0.26	2.24 ± 0.26	2.42 ± 0.33	6.01	0.000

*OFC*, occipitofrontal circumference.

*PI*, Ponderal Index.

Table 2 shows the mean maternal age, anthropometric indices and inter-pregnancy interval of symmetrical and asymmetrical SGA babies. The mean maternal age, weight, height, body mass index and the inter-pregnancy interval of the mothers with symmetrically small babies were lower than for mothers of asymmetrically small ones but the observed differences were not statistically significant.

**Table 2:** Mean maternal age, anthropometric indices and interpregnancy interval of symmetrical and asymmetrical small-for-gestational age babies

	**Symmetrical**	**Asymmetrical**		
	**Mean ± SD**	**Mean ± SD**	**t**	**p**
Maternal age (yrs)	28.24 ± 4.63	30.18 ± 4.55	1.61	0.11
Maternal weight (kg)	65.1 ± 15.63	71.4 ± 11.73	1.71	0.09
Maternal height (m)	1.55 ± 0.082	1.58 ± 0.064	0.49	0.63
Maternal BMI (kg/m^2^)	26.80 ± 4.86	28.60 ± 3.91	1.54	0.13
Inter-pregnancy				
Interval (yrs)	3.33 ± 3 42	2.33 ± 1.37	1.40	0.17

*BMI*, body mass index.

Using empirical cutoff values of maternal anthropometry, the associated frequency of symmetrically and asymmetrically small babies was compared in Table 3. There was no significant difference in the frequency of symmetrically small babies using the stated cutoff points for maternal weight (p = 0.15) and height (p = 0.51). However, increasing maternal BMI was associated with decreasing likelihood of symmetrically small babies but increasing likelihood of asymmetrically small babies.

**Table 3:** Frequency of symmetrical and asymmetrical SGA according to cutoff values of maternal anthropometry

	**N**	**Symmetrical**	**Asymmetrical**	***X***^**2**^	**p**
**Weight kg**					
< 60	17	10 (58.8)	7 (41.2)		
60 to 80	31	12 (38.7)	19 (61.3)	2.11	0.15
> 80	11	4 (36.4)	7 (63.6)		
**Height cm**					
< 155	39	16 (41.0)	23 (59.0)	0.43	0.51
≥ 155	20	10 (50.0)	10 (50.0)		
**Body mass index kg/m**^**2**^					
< 25	13	8 (61.5)	5 (38.5)		
25 to < 30	26	13 (50.0)	13 (50.0)	6.61	0.04
≥ 30	20	5 (25.0)	15 (75.0)		

Figures in brackets are percentages of N.

There was no significant influence of maternal parity on the clinical variety (symmetrical or asymmetrical) of SGA as shown in Table 4 (p = 0.41). Grandmultiparous mothers were however not included in the analysis because their number was too few for meaningful statistical analysis.

**Table 4:** Subgroups of SGA according to maternal parity

**Subgroup of SGA**					
**Maternal parity**		**Symmetrical**	**Asymmetrical**	***X***^**2**^	**p**
1	(n = 35)	14 (40.0)	21 (60.0)		
2 to 4	(n = 22)	11 (50.0)	11 (50.0)	0.68*	0.41
5+	(n = 2)	1 (50.0)	1 (50.0)		
Total	(n = 59)	26 (44.1)	33 (55.9)		

Figures in brackets are percentages of n.

* Comparison was between Para 1 and Para 2 to 4.

Table 5 show that the mothers of approximately 50% of symmetrically small as well as asymmetrically small babies did not experience significant morbidity in pregnancy (53.8% Vs 48.5%, p = 0.68). However, mothers of asymmetrically small babies had a significantly higher likelihood of experiencing hypertensive disease than their counterparts who had symmetrically small babies (*χ*^2^ = 5.71, p = 0.017: Risk ratio = 2.85 95% CI = −2.11 to 0.009).

**Table 5:** Relationship between maternal illness in pregnancy and frequency of sub-classes of small-for-gestational age babies

	**Symmetrical SGA**	**Asymmetrical SGA**	**All SGA**
	**N = 26**	**N = 33**	**N = 59**
Illness in pregnancy	No affected (%)	No affected (%)	No affected (%)
*Hypertensive disease	3 (11.5)	13 (39.4)	16 (27.1)
HIV infection	3 (11.5)	2 (6.1)	5 (8.5)
Sickle cell anaemia	2 (7.7)	2 (6.1)	4 (6.8)
Anaemia	2 (7.7)	0 (0.0)	2 (3.4)
Uterine fibroids	1 (3.8)	0 (0.0)	1 (1.7)
Achondroplasia	1 (3.8)	0 (0.0)	1 (1.7)
Malaria	0 (0.0)	1 (3.0)	1 (1.7)
No illness	14 (53.8)	16 (48.5)	30 (50.8)

Figures in brackets are percentages of N.

*Hypertensive disease comprises pre-eclampsia / eclampsia, pregnancy-induced hypertension, essential hypertension.

The pattern of PI in the subclasses of SGA babies is shown in Table 6. Of the 59 SGA babies, 44 (74.6%) had PI between the 10^th^ and 90^th^ percentiles while the remaining 15 (25.4%) had PI below the 10^th^ percentile. Of the 26 symmetrically small babies, 24 (92.3%) had PI within the normal range while two (7.7%) had values below the 10^th^ percentile. On the contrary, of the 33 asymmetrically small babies, 13 (39.4%) had low PI while 20 (60.6%) had PI within the normal range.

**Table 6:** Relationship between various combinations of growth restriction and Ponderal Index

			**Normal PI**	**Low PI**
**Restricted growth parameters**	**Subclass of SGA**	**N**	**no (%)**	**no (%)**
Weight, length and OFC	Symmetrical	1	1 (100.0)	0 (0.0)
Weight and length alone	Symmetrical	25	23 (92.0)	2 (8.0)
Weight alone	Asymmetrical	33	20 (60.6)	13 (39.4)

Figures in brackets are percentages of N.

## Discussion

The prevalence of 7.2% for small-for-gestational age obtained in the current study was similar to the 8.8% obtained in Harare, Zimbabwe [17] but lower than 12.8% and 12.01% in two earlier Nigerian studies in Sagamu [5] and Ilesa [6] respectively. All the three Nigerian studies under comparison (Sagamu, Ilesa and Lagos) emanated from tertiary hospital settings in the same southwestern part of Nigeria and it is unlikely that the quality of prenatal care in these centres varied remarkably. Therefore, the observed differences in the prevalence rates of SGA can be attributed to the fact that Sagamu and Ilesa communities are relatively less-urbanized and are occupied by less educated and less affluent population compared to Lagos, a highly industrialized city [5,6]. Furthermore, 70.4% of the study population in Lagos belonged to the upper socio-economic classes I and II compared to 19.5% in the Sagamu [5] and 15.7% in Ilesa [6].

On the other hand, the prevalence rate of SGA in the current study is much higher than 2.8 to 3.4% reported earlier in Sweden [3] and 4.1% in the United Kingdom. [4] The explanation may be found in the wide disparity in the socioeconomic development and the quality of health care between Nigeria and these other countries of the developed world. Generally, socioeconomic and nutritional problems as well as preventable diseases are key contributors to reduced birth weight in developing countries. [5,17] While none of the mothers in the current study agreed to alcohol consumption or cigarette-smoking, SGA deliveries in the developed world are largely accounted for by adverse social habits such as cigarette-smoking and alcohol consumption. [18-23].

The slight predominance of asymmetrically grown SGA babies in the current study (55.9%) conformed to earlier report from Sagamu [5] in which 58.3% of SGA were asymmetrical. This is probably a reflection of the short duration of the common types of intrauterine insults leading to restricted intra-uterine growth and SGA status in the population studied. Malaria, an endemic problem in Africa and indeed, in the aforementioned Nigerian populations, occurring in pregnant women may explain the relative predominance of asymmetric SGA since the illness typically resolves in a few days, when it is properly managed.

The pattern of PI observed within the subclasses of SGA babies in the current study tallied with expectation to varying extents. In conformity with conventional wisdom, all but two of the infants with symmetrical SGA had ponderal indices within the normal range. This is because PI is a mathematical ratio of weight-to-length and symmetrical reduction in both measurements will keep the ratio fairly unaffected. The two subjects in whom PI was low probably represent those in whom restriction in weight was more profound than that in length.

The observation with respect to asymmetric SGA babies was markedly different. Contrary to expectation, only 39% of subjects in this class had low PI while 61% had normal PI. This is similar to the report from Sagamu [5] in which as many as 71% of asymmetrical SGA babies had normal PI contrary to expectation. The finding therefore can neither be ignored nor easily dismissed. It may be a reflection of the relative effects of duration of intrauterine insult on weight and length. PI relies on the principle that length is spared at the expense of weight during periods of malnutrition. [24] As the duration of insults gets longer, length velocity gets more and more reduced. If the insult lasts long enough, weight and length velocities may be proportionally impaired and PI becomes ‘normal’. The implication is that intrauterine insults of relatively brief duration would be associated with reduced weight achievement, unaffected length achievement and therefore, reduced PI. On the other hand, insults of long duration would have had time to exert negative effects on both weight and length achievements, leading to normal PI. It is plausible therefore that insults of duration between the classically long and the classically short would produce varying degrees of reduction in length achievement and therefore varying values of PI from low to normal. This is in keeping with the description of body proportionality in SGA fetuses as a continuum [25]. Indeed, Davies *et al.*[26] used the terms ‘wasted’ and ‘not-so-wasted’ to allude to a continuum of intrauterine under-nutrition from the severely affected to those much less affected. Unfortunately, there are no other comparable Nigerian studies to support or refute this observation as representing a national pattern. Future studies from other Nigerian centers, possibly of a collaborative nature, might be useful in clarifying the true situation.

A number of factors were analyzed with a view to identifying those that may discriminate between symmetrical and asymmetrical SGA. Infant’s gender, family socioeconomic status and mean values of maternal size did not play a significant role. This pattern of observation was made by earlier workers in Sagamu. [5] It is apparent therefore that the role of factors like socioeconomic status and maternal size is limited to determining whether intrauterine growth restriction occurs or not and does not extend to the determination of the type of restriction. It would appear that determination of symmetry or otherwise of growth restriction is more of a function of duration of other specific adverse events. The two factors that discriminated significantly between varieties of SGA were maternal BMI and the prevalence of maternal hypertensive disease, with affected mothers being more likely to have the asymmetrical type.

Empirical cut-off of higher figures were used in this study for the reason of generally higher values obtained compared to the Ilesa and Sagamu studies. Maternal BMI of 22.7 kg/m^2^ for symmetrically small babies versus 22.2 kg/m^2^ for asymmetrically small babies were recorded in Sagamu [5] compared to 25.2 kg/m^2^ versus 26.5 kg/m^2^ in Ilesa[27] and 26.8 kg/m^2^ versus 28.6 kg/m^2^ in Lagos. The weights and heights of the mothers in the Sagamu [5] and Ilesa [27] studies were also generally lower compared to the values obtained in Lagos. Nevertheless, Dawodu and Laditan [28] in the same urban community adopted heights of less than 155 cm as cut off which was used in the current study. With respect to BMI, the increasing maternal BMI associated with decreasing likelihood of symmetrically small babies and increasing likelihood of asymmetrically small ones was a reflection of the association between maternal nutritional status and intra-uterine growth. This is in consonance with findings in some previous studies. [5,27] Low maternal BMI is used as evidence of maternal malnutrition (thinness) [29] and low maternal BMI is caused by chronically low energy intake at the household level [29,30]. The general implication is that effort should be made to improve food security in the population studied and ultimately, improve the nutritional status of women of child-bearing age in the general community and a vigorous pursuit of food supplementation and health education programmes should be very useful in this regard.

Hypertension on the other hand has been implicated in the aetiology of asymmetrically restricted babies in earlier studies. [31] The plausible explanation is probably related to time of outset and duration of hypertensive disease before delivery. Hypertension beginning late in pregnancy would expectedly affect fetal weight than length as a result of the short duration of exposure of the fetus to associated placental insufficiency. [9,31] Also, obstetricians are more likely to intervene relatively early with caesarean delivery in cases of severe hypertension occurring close to term, thus reducing the period of fetal exposure to placental insufficiency. The implication is that even if the weight is adversely affected, growth in length is relatively spared and therefore the baby will be classified as asymmetrical. This is the basis to improve antenatal screening and prompt treatment of hypertension in pregnancy.

The expectation of ‘normal’ PI in symmetrical SGA babies was met in 92.3% of cases in the current study. On the other hand, the expectation of low PI in asymmetrical SGA babies was only met in 39.4% of cases. The finding supports the opinion that proportionality in SGA fetuses is a continuum with the PI depending on the duration of intra-uterine insult and the extent of its effects on weight and length before delivery.

## Conclusion

A continuum of PI was found from normal values (mostly in symmetrical SGA babies) to low values found in both symmetrical and asymmetrical SGA babies, probably reflecting varying durations of intrauterine insult. Only maternal BMI and hypertensive disease significantly differentiated between symmetrical and asymmetrical SGA babies in the present study. Maternal hypertensive disease in pregnancy was significantly more often associated with asymmetrical than symmetrical SGA. Maternal age, parity and anthropometry did not significantly differ between the subclasses of SGA babies.

## Competing interests

The authors’ declare that they have no competing interests.

## Authors’ contributions

All authors read and approved the final manuscript.

## Pre-publication history

The pre-publication history for this paper can be accessed here:

http://www.biomedcentral.com/1471-2431/13/110/prepub
